# Regulation of the H^+^-ATP synthase by IF1: a role in mitohormesis

**DOI:** 10.1007/s00018-017-2462-8

**Published:** 2017-02-06

**Authors:** Pau B. Esparza-Moltó, Cristina Nuevo-Tapioles, José M. Cuezva

**Affiliations:** Departamento de Biología Molecular, Centro de Biología Molecular Severo Ochoa, (CSIC-UAM), Centro de Investigación Biomédica en Red de Enfermedades Raras CIBERER-ISCIII, Instituto de Investigación Hospital 12 de Octubre, Universidad Autónoma de Madrid, 28049 Madrid, Spain

**Keywords:** Mitochondria, ATPase inhibitory factor 1 (IF1), Metabolic reprogramming, Reactive oxygen species (ROS), Cancer, Lifespan

## Abstract

The mitochondrial H^+^-ATP synthase is a primary hub of cellular homeostasis by providing the energy required to sustain cellular activity and regulating the production of signaling molecules that reprogram nuclear activity needed for adaption to changing cues. Herein, we summarize findings regarding the regulation of the activity of the H^+^-ATP synthase by its physiological inhibitor, the ATPase inhibitory factor 1 (IF1) and their functional role in cellular homeostasis. First, we outline the structure and the main molecular mechanisms that regulate the activity of the enzyme. Next, we describe the molecular biology of IF1 and summarize the regulation of IF1 expression and activity as an inhibitor of the H^+^-ATP synthase emphasizing the role of IF1 as a main driver of energy rewiring and cellular signaling in cancer. Findings in transgenic mice in vivo indicate that the overexpression of IF1 is sufficient to reprogram energy metabolism to an enhanced glycolysis and activate reactive oxygen species (ROS)-dependent signaling pathways that promote cell survival. These findings are placed in the context of mitohormesis, a program in which a mild mitochondrial stress triggers adaptive cytoprotective mechanisms that improve lifespan. In this regard, we emphasize the role played by the H^+^-ATP synthase in modulating signaling pathways that activate the mitohormetic response, namely ATP, ROS and target of rapamycin (TOR). Overall, we aim to highlight the relevant role of the H^+^-ATP synthase and of IF1 in cellular physiology and the need of additional studies to decipher their contributions to aging and age-related diseases.

## Introduction

Mitochondria have been traditionally regarded as the “power stations” of the cell since they produce most of cellular energy requirements in a process known as oxidative phosphorylation (OXPHOS). They are also primary hubs of intermediary metabolism because they generate precursors for the synthesis of macromolecules and synthesize heme and Fe-S clusters present in some proteins. However, this notion has been expanded in recent years after the demonstration of the role played by mitochondria in the execution of cell death [1–3] and in establishing pathways to communicate their status to the nucleus [4, 5] and to other compartments [6] to adapt cellular responses to different programs, such as proliferation, differentiation and stress responses [4, 7]. Mitochondrial communication is exerted by signaling molecules that emanate from the organelle and affect by different mechanisms cell fate and behavior. Some of the signaling molecules include Ca^2+^, reactive oxygen species (ROS), metabolites, such as citrate, ATP, phosphatidylethanolamine, and mitochondrial resident proteins and/or peptides encoded in mtDNA [7–15].

The dynamic fusion and fission events experienced by the mitochondrial reticulum are also involved in signaling [16–18]. The primary sensor of mitochondrial status is the mitochondrial membrane potential (ΔΨm) and preventing its collapse is essential for cellular viability [19]. The mitochondrial signalosome primarily triggers the reversible and/or irreversible modification of proteins and/or the allosteric regulation of enzymes that result in protein activation/inactivation of particular genetic and/or epigenetic programs for cellular adaptation. The list of programs affected by the mitochondrial signalosome is just being glimpse, and remarkably, some of them are responsible for promoting adaptive mechanisms that allow cells to withstand subsequent detrimental stresses, a concept coined as “mitohormesis” [20, 21].

A primary hub of mitochondrial energy generation and signaling is the OXPHOS system that integrates the complexes of the electron transport chain (ETC) and the H^+^-ATP synthase in defined supercomplexes and subdomains of the inner mitochondrial membrane (IMM) [22–24]. In OXPHOS, the electrons derived from biological oxidations are transferred to the complexes of the ETC to reduce molecular oxygen and to generate the proton gradient used for the synthesis of ATP by the H^+^-ATP synthase [25, 26]. Even under conditions of efficient electron transfer a small percentage of the electrons funneled into the ETC generate superoxide radical [9], which is rapidly converted into the signaling molecule hydrogen peroxide. Its production is largely increased in pathophysiological situations [7, 27, 28] or could contribute to mitohormesis [20, 21]. This review is mainly focused on the regulation of the activity of the H^+^-ATP synthase by its physiological inhibitor the ATPase inhibitory factor 1 (IF1) with emphasis in the different signaling pathways that contribute to mitohormesis as a result of its activation as inhibitor of the engine of OXPHOS.

## The H^+^-ATP synthase: a core hub in mitochondrial structure and function

**Fig. 1:**
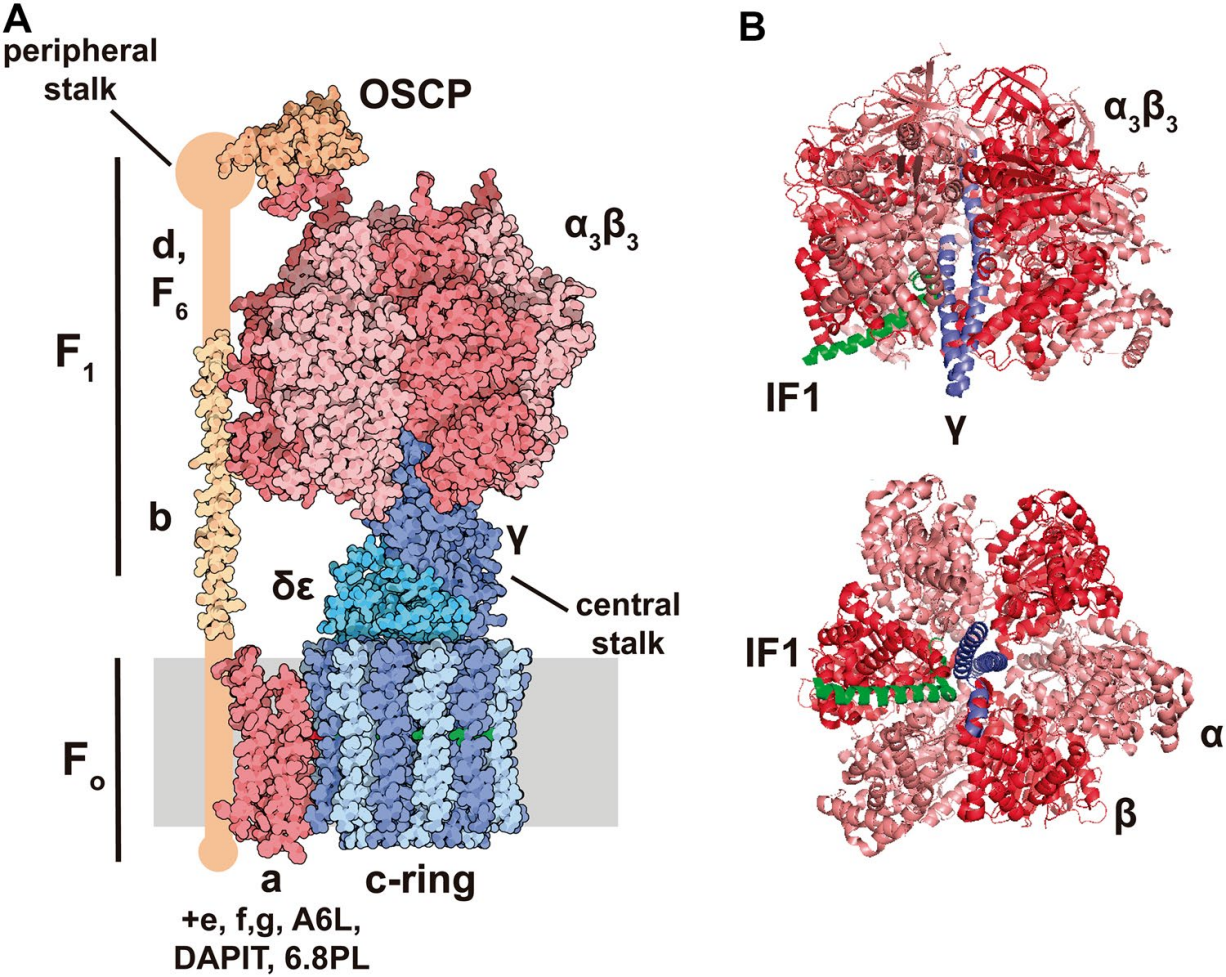
Structure of the bovine H^+^-ATP synthase and binding site of IF1. **a** The soluble F_1_-ATPase domain is composed by the 3α3β subassembly (*salmon*/*red*) and γ (*dark blue*), δ and ε (*light blue*) subunits, while the membrane-embedded F_o_ domain is formed by subunit a (*red*) and a ring of 8c subunits (*light*/*dark blue*). Both domains are linked together by a central stalk (γ, δ, ε subunits of the F_1_ domain) and a peripheral stalk (b, d F6, A6L, OSCP subunits, in *orange*). The 3D structure of the peripheral stalk is not fully resolved. Except for a and A6L subunits, the remainder are encoded in the nucleus. Molecular reconstruction from PDB. **b** Lateral and basal view of the bovine F_1_ domain (α subunit is shown in salmon, β in *red* and γ in *blue*) complexed with a fragment of IF1 (*green*). IF1 binds to the αβ interface through residues 1–37 and also contacts the γ subunit. Images taken from [44] (PDB: 1OHH) and created with the PyMOL Molecular Graphics System

The H^+^-ATP synthase is a multisubunit complex built by two major functional domains: the membrane-embedded F_o_-ATPase (a and 8c subunits) and the membrane-extrinsic catalytic F_1_-ATPase (3α, 3β, γ, δ and ε subunits) domains [25] (Fig. 1a). Both domains are linked by a central stalk that contains γ, δ and ε subunits of the F_1_-domain and a peripheral stalk that contains b, d, F6, and OSCP (oligomycin sensitivity-conferring protein) subunits [25] (Fig. 1a). Additional subunits have been described in the mitochondrial complex when the enzyme is purified in the presence of phospholipids: e, f, g, A6L, DAPIT (diabetes-associated protein in insulin-sensitive tissues) and the 6.8-kDa proteolipid (6.8PL). Their role in the activity of the enzyme remains to be fully characterized [25, 29]. ATP synthesis by the H^+^-ATP synthase is driven by the influx of protons into the matrix through the a subunit that promotes the rotation of the c-ring and of the attached central stalk, the later triggering the conformational changes in the F_1_-ATPase that drives ATP synthesis from ADP and Pi [25, 30]. The H^+^-ATP synthase is a reversible engine because ATP hydrolysis triggers rotation of the c-ring in the opposite direction generating a proton gradient across the inner membrane [25, 30].

The assembly of the H^+^-ATP synthase in *Saccharomyces cerevisiae* comprises two separate and coordinated pathways that involve (1) the stepwise assembly of Atp6 (the homologue of a subunit) and Atp8 (the homologue of A6L subunit) with the peripheral stalk and (2) the assembly of the c-ring with the F_1_-ATPase domain [31, 32]. Later steps in the assembly process are mediated by the INA complex, a matrix-exposed inner membrane protein complex that facilitates the assembly of the peripheral stalk [33]. The assembly process in humans is similar to yeast [34]. Two of the “supernumerary” subunits, DAPIT [35] and 6.8PL [36], seem to be involved in the assembly process because its silencing in HeLa cells causes the loss of H^+^-ATP synthase complexes. Super-assemblies of the H^+^-ATP synthase [24] play an important role in determining the structure of the mitochondrial inner membrane because they contribute to the generation of *cristae* [37]. In fact, dimeric arrays of the H^+^-ATP synthase in the inner membrane of yeast [38] and mammalian mitochondria [39] generate the curvature of the IMM that promotes cristae formation [37, 40]. Recent findings suggest that while the e–g heterodimer promotes the local membrane curvature, the f subunit in the monomers establishes the contacts in the dimer interface [41].

The ATPase inhibitory factor 1 (IF1), which is a main regulator of the activity of the H^+^-ATP synthase (see below) has also been claimed to promote dimerization of the H^+^-ATP synthase [42–47]. However, other studies do not support this idea [48–50] or suggest that IF1 is just a bridge stabilizing two F_1_-ATPase domains with no crucial role in dimer formation [51]. Interestingly, recent findings support that the mitochondrial permeability transition pore (PTP) [3] contains dimers of the H^+^-ATP synthase [3, 52–54], consistent with the finding that subunit c of the F_o_ domain is required for the activity of the PTP [55, 56]. Interestingly, it has been suggested that the low probability of PTP opening at low pH might be due to the binding of IF1 [3] because the histidine reagent diethylpyrocarbonate is able to restore the ability to induce PTP opening and prevents IF1 binding to the inner membrane [3]. However, alternative explanations for IF1-mediated protection against cell death have already been provided in cellular [57] and in vivo mouse models [42, 58] by the mitohormetic role played by IF1.

The activity of the H^+^-ATP synthase is regulated by the cellular energy demand, the covalent modification of several of its subunits and by different metabolites and regulatory proteins that interact with the enzyme [59–62]. One of the key regulators of the H^+^-ATP synthase activity is Ca^2+^ [8]. Mitochondria are crucial organelles in intracellular Ca^2+^ buffering and signaling [63, 64]. S100A1 is a Ca^2+^-sensing protein that is expressed predominantly in cardiac muscle and interacts with the F_1_-ATPase in a Ca^2+^-dependent manner, increasing its activity [65]. Consistently, S100A1 knockout mice show reduced H^+^-ATP synthase activity in cardiomyocytes [65]. The Ca^2+^-inhibitor binding protein (CaBI) is another Ca^2+^-sensitive protein that dissociates from the H^+^-ATP synthase molecule in the presence of high intracellular Ca^2+^ levels and has been suggested as a candidate for the regulation of the H^+^-ATP synthase [61, 66].

The major Ca^2+^ store in the cell is the endoplasmic reticulum (ER). Contacts between mitochondria and the ER called mitochondria-associated membranes (MAMs), mediate the transfer of ions, metabolites, proteins and phospholipids, including Ca^2+^ [6]. One important complex involved in these contacts is the yeast ERMES (ER-mitochondria encounter structure) complex [67], which is composed by four core proteins resident both in the ER and in mitochondria. These proteins are functionally connected to phospholipid biosynthesis and Ca^2+^ signaling [67]. ERMES is naturally involved in establishing and maintaining contact sites between the two organelles [68]. Further studies have revealed that ERMES is involved in mitochondrial dynamics, inheritance, protein import, mtDNA inheritance and mitophagy [69]. No orthologues of the ERMES complex have been found in mammals.

DAPIT, a “supernumerary” subunit of the H^+^-ATP synthase, may also play a role in the regulation of the H^+^-ATP synthase since it is highly expressed in cells with high aerobic metabolism [70]. Knockdown of DAPIT results in a drastic reduction in the activity and content of H^+^-ATP synthase complexes in human cells [35]. In contrast to these findings, the overexpression of DAPIT in HEK293T cells impairs mitochondrial biogenesis and promotes the generation of ROS, both resulting in an enhanced aerobic glycolysis and the activation of the Wnt/β-catenin signaling pathway [71].

Besides S100A1, CaBI and DAPIT, the activity of the H^+^-ATP synthase is regulated by additional interacting proteins, including the antiapoptotic protein Bcl-xL (B-cell lymphoma-extra-large) [72], the hypoxia-induced protein G0/G1 switch gene 2 (G0s2) [73] and coupling factor B (FB) [29]. Moreover, different covalent modifications and metabolites produced in mitochondria regulate the activity of the H^+^-ATP synthase (see [62] for details).

## The ATPase inhibitory factor 1 (IF1): a master regulator of the activity of the H^+^-ATP synthase

**Fig. 2:**
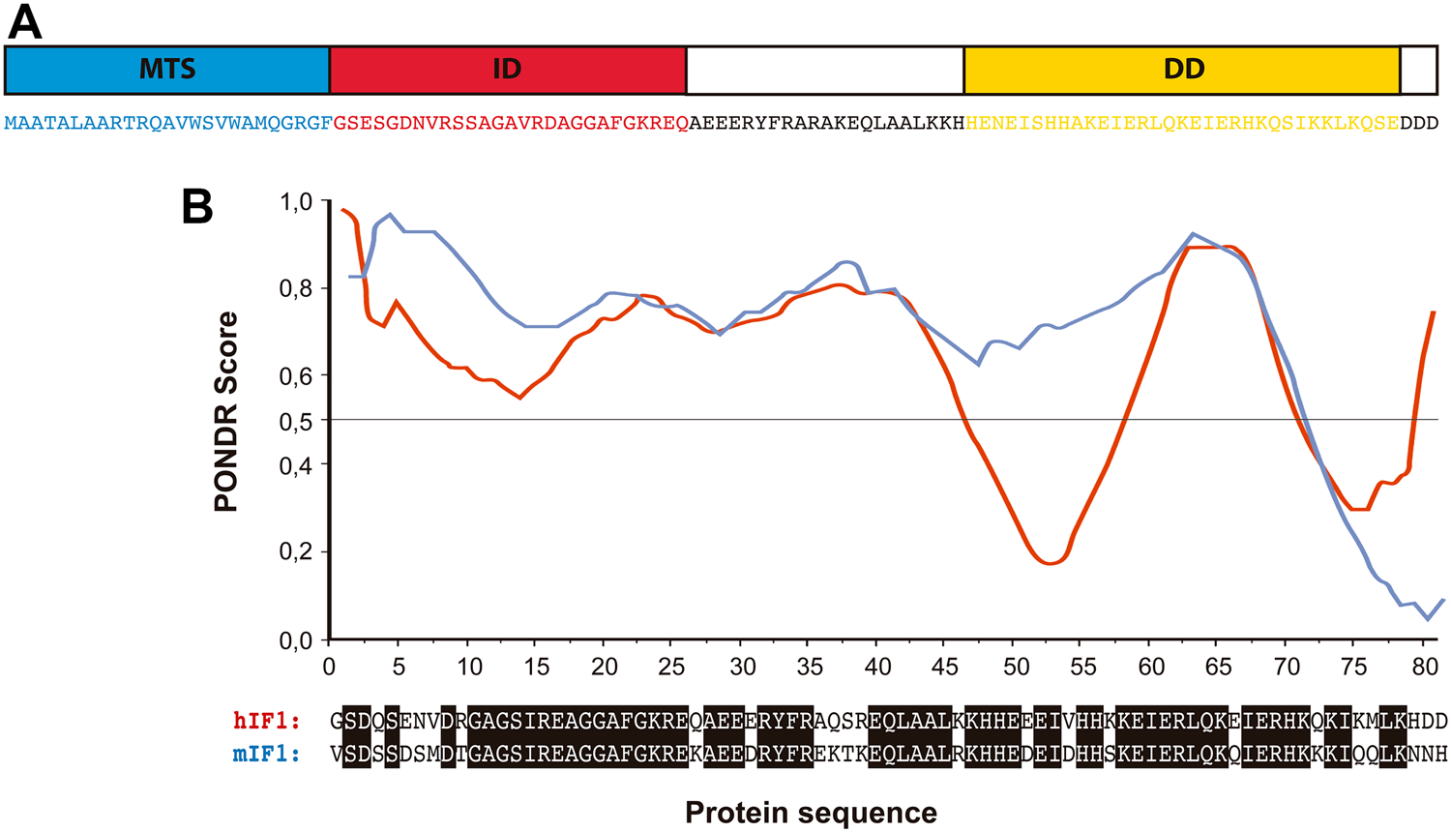
IF1 is an intrinsically disordered protein. **a** Amino acid sequence of bovine IF1 and scheme of the main domains of the protein: the mitochondrial targeting sequence (MTS, *blue*), the N-terminal inhibitory domain (ID, *red*) and the α-helical coiled-coil dimerization domain (DD, *yellow*) are highlighted. **b** Prediction of the disordered regions of human (hIF1) and mouse IF1 (mIF1) using Predictor of Natural Disordered Regions (PONDR®) VLXT algorithm. The regions with PONDR score >0.5 are predicted as disordered

IF1 was first described in mitochondria from bovine heart as a small protein that inhibits the soluble F_1_ domain of the H^+^-ATP synthase [74] (Fig. 1b) with homologs in plants, yeasts, animals and humans (for review see [62]). The mammalian proteins present a high degree of sequence conservation and are able to inhibit the H^+^-ATP synthase of different species, including yeast [75]. In humans, IF1 is encoded in the nuclear *ATPIF1* gene, located in chromosome 1, is translated as a precursor protein that upon mitochondrial import experiences the removal of the N-terminal 25-residue long pre-sequence [62]. The mature protein comprises a N-terminal intrinsically disordered domain [43] that interacts with the H^+^-ATP synthase and a C-terminal dimerization domain [76] (Fig. 2a). Bioinformatic predictions suggest that human and mouse homologues are also intrinsically disordered proteins (Fig. 2b). Intrinsically disordered proteins are important components of cellular signaling, since they can respond to intra- or extracellular cues by disorder-to-order conformational changes to exert a plethora of outcomes [77]. IF1 binding site is located in the catalytic interface between the α and β subunits of the F_1_ domain (Fig. 1b) as inferred using a truncated form of bovine IF1 (I1-60His) [78]. During the binding process, the N-terminus of I1-60His changes to an ordered α-helical structure [79] and once bound, IF1 blocks the rotation of the complex inhibiting the hydrolysis of ATP [80] (Fig. 1b).

**Fig. 3:**
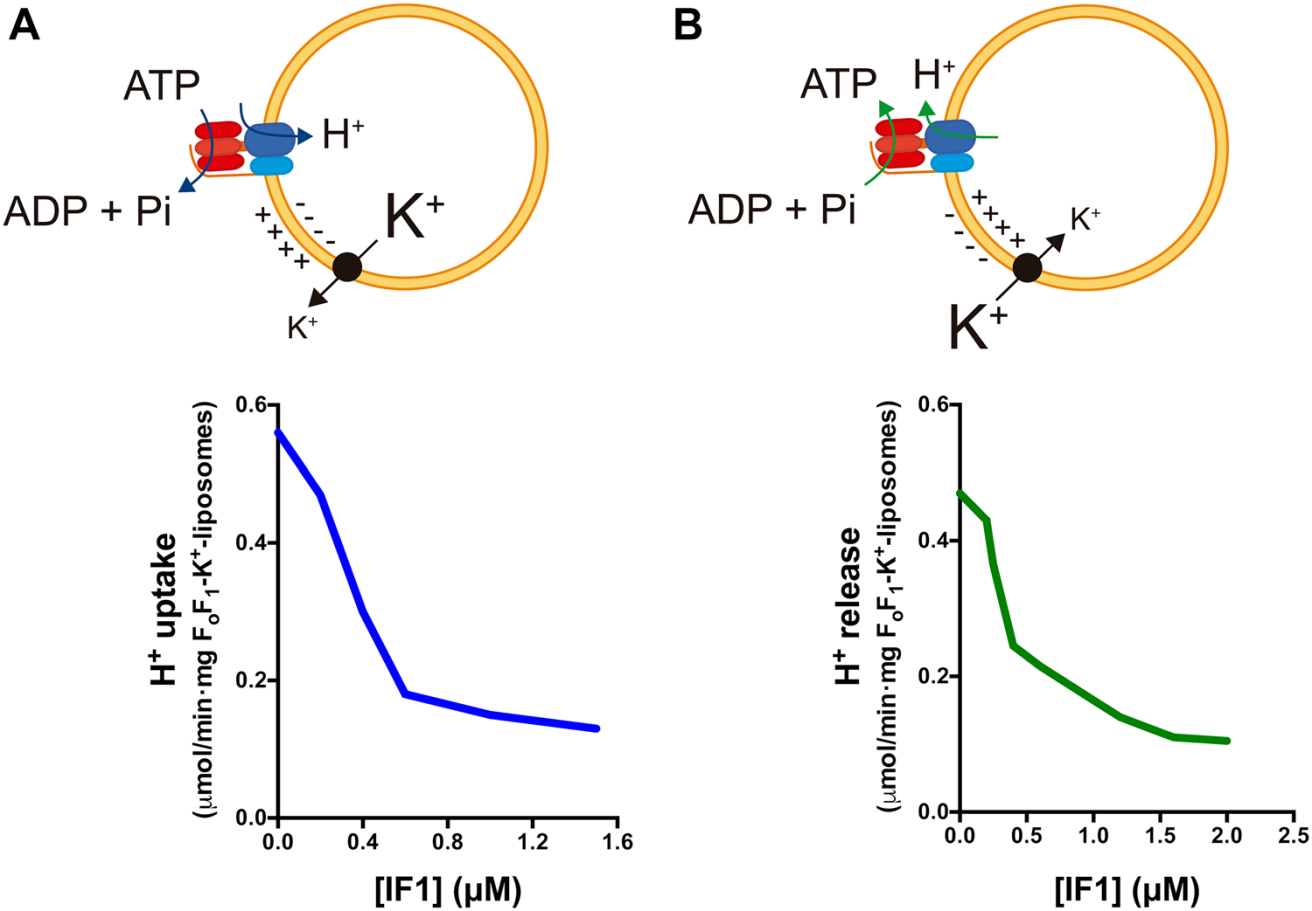
IF1 inhibits H^+^ translocation through the H^+^-ATP synthase in both synthetic and hydrolytic modes. **a** H^+^ uptake is induced by valinomycin-mediated K^+^ release from F_o_F_1_-K^+^ liposomes with the H^+^-ATP synthase functioning in the hydrolytic mode. **b** H^+^ release is induced by valinomycin-mediated K^+^ uptake in F_o_F_1_-K^+^ liposomes with the H^+^-ATP synthase functioning in the synthetic mode. Rates of H^+^ uptake **(a)** and H^+^ release **(b)** are reduced when the liposomes are incubated with increasing concentrations of isolated IF1. *Black circle* valinomycin. Adapted from [84]

Recent findings point out that IF1 when bound to the enzyme inhibits both the ATP hydrolase and synthase activities [81] rather than acting solely as a unidirectional inhibitor of the hydrolase activity of the H^+^-ATP synthase [25, 46]. Indeed, inhibition of the H^+^-ATP synthase activity has been reported by the overexpression of IF1 or its constitutively active mutant H49K in cells in culture [57, 82, 83] and in neurons [58] and in hepatocytes [42] of transgenic mice in vivo. Consistently, it has been reported that IF1 inhibits the translocation of protons mediated by the H^+^-ATP synthase when operating in synthetic and hydrolytic modes [84] (Fig. 3). The IF1-mediated inhibition of the ATP synthetic activity can also be traced by the activation of signaling pathways sensing energy deprivation and the subsequent activation of glycolysis [42, 57, 58, 82, 83]. More direct confirmation that IF1 inhibits both activities of the enzyme was recently provided after showing that its binding to the synthase and inhibitory activities depend on its phosphorylation status [81].

## Regulation of IF1 activity and expression

**Fig. 4:**
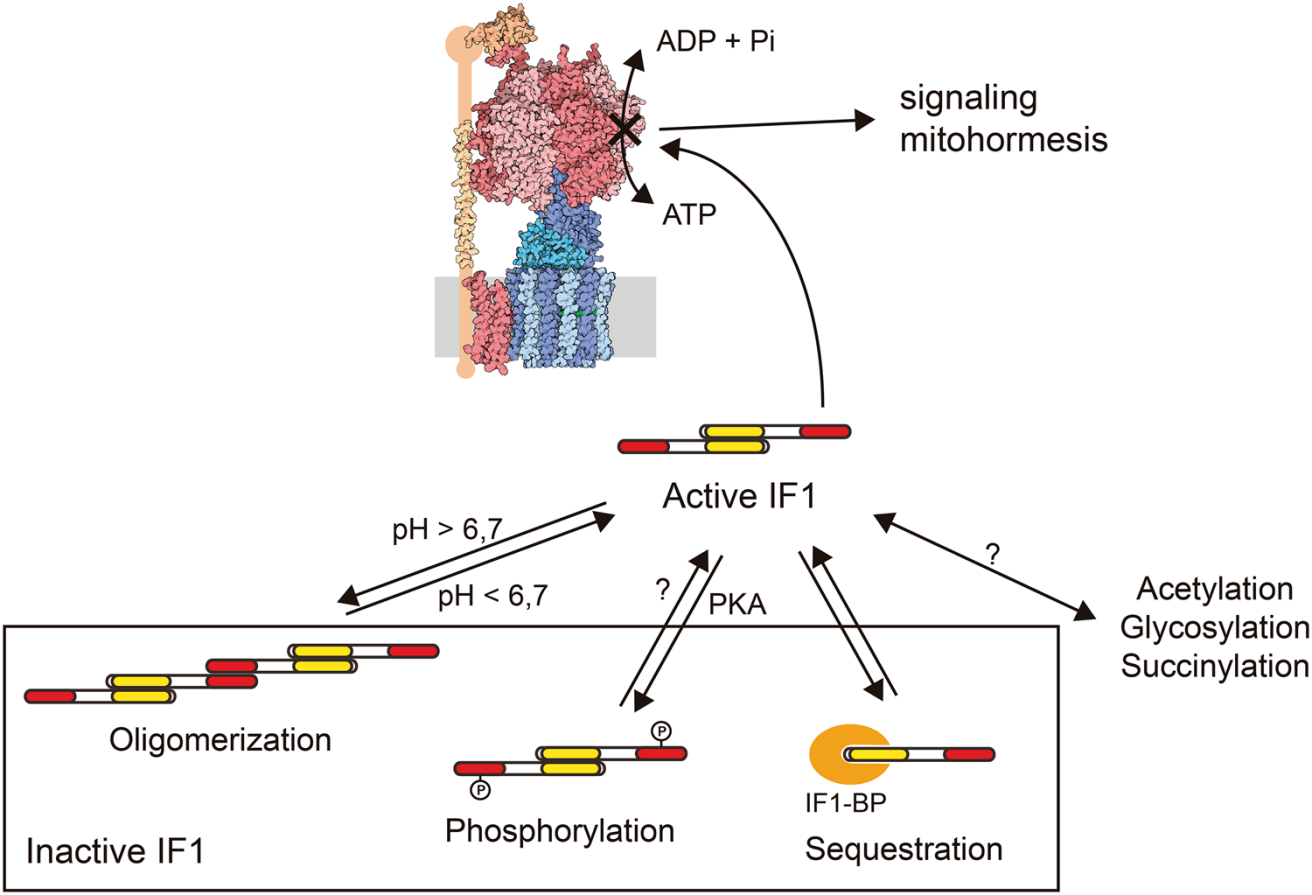
Post-translational regulation of IF1 activity. IF1 can be inhibited by pH-dependent oligomerization, phosphorylation mediated by a mitochondrial cAMP-dependent protein kinase A-like activity (PKA) or by sequestration by a mitochondrial membrane protein (IF1-BP). IF1 is also the substrate for acetylation, glycosylation and succinylation, although the physiological effects of these modifications remain to be fully characterized. The pool of active IF1 determines the population of inhibited H^+^-ATP synthase complexes, which might trigger retrograde signaling pathways

The regulation of IF1 activity as an inhibitor of the hydrolase activity of the H^+^-ATP synthase involves five histidine residues whose ionization status play an active role in the structure and oligomerization of IF1 [85]. When the mitochondrial matrix acidifies, i.e., when mitochondria become de-energized, such as in hypoxia and ischemia, IF1 tends to form inhibitory dimers [85]. At pH higher than 6.7, IF1 tends to form inactive tetramers by the interaction of two dimers through the N-terminus of the protein, hence masking the region that binds to the H^+^-ATP synthase [76, 85] (Fig. 4). Site-directed mutagenesis of H49 into a lysine generates the IF1-H49K mutant, which is active as inhibitor of the enzyme at pH higher than 6.7 [85] and has been used to effectively inhibit OXPHOS in a tissue-specific manner in transgenic mice [42, 58].

More recently, we have demonstrated that the inhibitory activity of IF1 depends on the phosphorylation status of a conserved serine 39 (S39) [81]. In the dephosphorylated state, IF1 binds to the H^+^-ATP synthase and inhibits both activities of the enzyme (Fig. 4). Phosphorylation of IF1 prevents its interaction with the H^+^-ATP synthase, abolishing its inhibitory activity (Fig. 4). IF1 is phosphorylated in vivo by the activity of a mitochondrial cAMP-dependent protein kinase [81]. Actually, PKA regulates diverse mitochondrial functions [62, 86], including an increase in the efficiency of OXPHOS [87–90].

A soluble adenylyl cyclase (sAC) has been described inside mitochondria [87, 91, 92], which is activated by Ca^2+^ [89] and bicarbonate [87]. In this regard, it is interesting to note that β-adrenergic stimulation of mouse heart in vivo also promotes an increase in cAMP levels inside mitochondria, the phosphorylation of IF1 and the subsequent activation of the H^+^-ATP synthase boosting ATP supply by OXPHOS [81]. We have recently summarized other physiologically relevant contexts for the regulation of IF1 activity by phosphorylation for tuning substrate availability and energy demand with OXPHOS activity in hypoxia, cell cycle progression and cancer [62, 81]. The mechanisms regulating the dephosphorylation of IF1 (if any) are unknown [62]. Nevertheless, we have suggested that the short half-life of the protein [83, 93] may rapidly reestablish the content of active dephospho-IF1 inside the organelle.

Besides the canonical binding site on the H^+^-ATP synthase, IF1 can also bind to OSCP [94] and to a small mitochondrial membrane protein that hampers its activity as an inhibitor of the H^+^-ATP synthase [95] (Fig. 4). Hence, IF1 oligomerization, phosphorylation and binding to a putative “receptor” may regulate the pool of active IF1 in mitochondria, allowing OXPHOS to adapt to different conditions of substrate availability and energy demand (Fig. 4). Moreover, additional covalent modifications of IF1 have been reported (Fig. 4) but their physiological relevance remains to be investigated [62].

Table 1 summarizes and compares the expression level of IF1 in different human tissues. As we have observed in mouse heart [81], not all the IF1 present in the tissue is active as an inhibitor under basal physiological conditions [81]. In fact, and based on the findings in heart, we suggest that a large fraction of IF1 is maintained functionally inactive (Fig. 4) in high energy-demanding tissues that show a high content of IF1 (brain, kidney and liver) to provide a reservoir of inhibitor that could prevent ATP wasting when OXPHOS is compromised [81]. On the other hand, the fraction of active IF1 inhibitor offers a pool of inactive H^+^-ATP synthase in the tissue that upon an enhanced metabolic demand could respond increasing the supply of ATP after the inactivation of IF1 (Fig. 4) [81]. We suggest that this pool of inactive H^+^-ATP synthase might contribute to retrograde signaling [62, 81] (Fig. 4).

**Table 1:** IF1 expression in normal human tissues and in the corresponding carcinomas

Organ/tissue	Normal	Carcinoma	Prognosis (High IF1)	References
Bladder	Negligible	High	High-risk	[107]
Brain (Neurons)	High	–	–	[58]
Brain (Glia)	Negligible	High	High-risk	[58, 113]
Breast	Negligible	High	Low-risk	[83]
Colon	Negligible	High	Low-risk	[57, 83, 109]
Endometrium	High	High	–	[83]
Kidney	High	High	–	[83]
Liver	High	High	High-risk	[83, 112]
Lung	Negligible	High	High-risk	[83, 108]
Ovary	Negligible	High	–	[83]
Stomach	High	High	High-risk/low-risk	[83, 109, 111]

IF1 expression level is defined as negligible or high as assessed in western blots and/or immunohistochemistries. The relevance of IF1 as biomarker of survival and/or of disease recurrence was assessed in Kaplan–Meier plots using the cutoff to define potentially “high-risk” and “low-risk” groups as indicated in the indicated references. A high expression level of IF1 stratifies the cohort of patients as indicated. With the exception of data in reference [109] that has used mRNA levels, the rest of the studies used protein IF1 expression data

## IF1 in cancer

Down-regulation of OXPHOS and the concurrent activation of aerobic glycolysis is a hallmark of cancer [96] and of undifferentiated cells [93, 97], including induced pluripotent stem cells (iPSCs) [98]. This metabolic phenotype is optimal because it supplies the anabolic intermediates needed for proliferation [99–101], and also protects from mitochondrial derived ROS [102] and cell death [103]. The analysis of large cohorts of different human carcinomas indicated that the partial reduction of OXPHOS in cancer cells is achieved by down-regulation of the expression of the catalytic subunit of the H^+^-ATP synthase (β-F1-ATPase) [104–106] (for additional references in other carcinomas see [99]). More recently, we have shown that metabolic rewiring to an enhanced glycolysis is also exerted by the overexpression of IF1 in carcinomas [57, 82, 83] (Table 1). Remarkably, the IF1 present in these carcinomas is found in its active dephosphorylated state [81].

Table 1 shows the changes in IF1 expression levels observed between normal tissues and the corresponding carcinomas. Tissues, such as bladder, breast, colon, lung and ovary show negligible expression level of IF1 under normal physiological conditions [57, 83, 107] (Table 1). However, upon transformation, the carcinomas arising in these tissues show very high levels of IF1 (Table 1). Interestingly, whereas in bladder [107] and non-small cell lung carcinomas [108] a high expression level of IF1 in the tumor predicts a worse patients’ prognosis, a low tumor expression of IF1 predicts a worse prognosis in colon and breast cancer patients [83]. Consistently, a recent study using mRNA expression levels also suggests that a high IF1 expression predicts the group of low-risk patients in colon cancer [109]. In agreement with a higher metastatic potential of breast cancer cells expressing a low level of IF1, it has been reported that lymph node metastasis of breast cancer patients had a lower expression level of IF1 when compared to the primary tumors [110]. The reasons for the differential behavior of IF1 in cancer progression in these carcinomas remain to be investigated.

High energy-demanding human tissues, such as brain, liver and kidney, and in addition stomach and endometrium, express the highest levels of IF1 under normal physiological conditions [82, 83] (Table 1). For most of these tissues (endometrium, kidney, liver and stomach) carcinogenesis does not promote a relevant increase in the tumor expression level of IF1 [83] (Table 1). However, in gastric carcinomas [111] and in hepatocarcinomas [112] the tumors that show a higher expression level of IF1 predict a worst patient prognosis. However, a recent study using mRNA expression levels suggests that a high IF1 expression predicts the group of low-risk patients [109]. Especial consideration deserves the brain which shows very high expression levels of IF1 in neurons and negligible in astrocytes [58] (Table 1). Interestingly, gliomas show a very high expression of IF1 that correlates with a poor patient prognosis [113] (Table 1).

The *ATPIF1* proximal promoter contains a NFκB (nuclear factor kappa B) binding site [83]. It has been reported that binding of this transcription factor activates *ATPIF1* transcription in a feedback loop that promotes hepatocarcinogenesis [112]. However, the accumulation of IF1 protein in other prevalent human carcinomas (colon, lung, breast and ovary) occurs with no relevant changes in *ATPIF1* mRNA levels [83], suggesting that IF1 expression is mainly regulated at post-transcriptional levels in agreement with findings in other systems [114]. Indeed, IF1 has a very short half-life (~2–4 h) [83] and the control of its turnover is a relevant event in the differentiation of human mesenchymal stem cells [93]. The degradation of IF1 may involve several mitochondrial proteases [115], including serine-proteases [83, 93] and metalloproteases [116]. Mouse Ier3 (immediate early response 3) has been shown to physically interact with the C-terminal domain of IF1 and promote its degradation in a proteasome-independent way that requires ATP [116].

IF1 overexpression triggers mitochondrial hyperpolarization and the production of superoxide radical [57, 82]. Superoxide is rapidly converted into hydrogen peroxide, a ROS signaling molecule that activates nuclear programs of cell death protection. Mechanistically, ROS-mediated signaling induces the canonical NFκB pathway in IF1-overexpressing cancer cells [57, 83] and in neurons [58] promoting Bcl-xL-guided cell death protection. The nuclear signaling pathways activated in response to IF1 overexpression vary depending on the tissue. In fact, in liver it seems that the antioxidant response guided by Nrf2 [nuclear factor (erythroid-derived 2)-like 2] is crucial in the protection of hepatocytes from oxidative insults [42]. However, we should mention that the metabolic pre-conditioning afforded by IF1 overexpression in mouse liver is deleterious in the context of hepatocarcinogenesis [42], in agreement with recent findings in humans [112].

Since the H^+^-ATP synthase is a critical component of the PTP, IF1 by potentially regulating the oligomeric state of the H^+^-ATP synthase, might contribute to upgrade at the structural level the threshold to execute cell death [58, 117]. However, the overexpression of IF1-H49K mutant in the liver of transgenic mice, which promotes the formation of H^+^-ATP synthase dimers and protects the hepatocytes from oxidative insults, indicated that this protection is unrelated to differences in PTP opening and regulation [42]. Hence, we suggest that the cell death protection afforded by IF1 is primarily exerted by signaling mitohormesis [42, 57, 58].

## Nucleo-mitochondria communication: mitohormesis

There is growing evidence supporting that a mild mitochondrial stress can protect the cell from subsequent insults, a concept termed mitohormesis [20, 21]. Mitohormesis is defined as an adaptive cellular response triggered by a mild mitochondrial malfunctioning that activates cytoprotective mechanisms to compensate the primary defect and results in long-lasting broad metabolic and molecular changes. These adaptive changes may finally lead to increased lifespan and healthspan. The mitohormetic response requires the existence of signaling pathways that sense mitochondrial function and inform the nucleus to trigger the adequate cellular programs to compensate stress [4]. The signaling pathways emanating from both mitochondria and the nucleus have been extensively reviewed elsewhere [4, 5].

A stress response pathway with well described cytoprotective effects is the mitochondrial unfolded protein response (UPR^mt^). Mitochondrial stress does not only involve a compromise in OXPHOS activity, arising from defects in mtDNA or poisoning of the ETC, but also by an accumulation of unfolded mitochondrial proteins [118]. Proteotoxic stresses that exceed the capacity of mitochondrial chaperones and proteases induce a transcriptional program in the nucleus in an attempt to restore proteostasis and adapt the cellular metabolism to the mitochondrial stress. This pathway has been thoroughly described in *Caenorhabditis elegans* [119–122] and is conserved in mammals [123–125]. In *C. elegans*, the sensor protein is ATFS-1 (activating transcription factor associated with Stress-1), which is normally imported into mitochondria and degraded in the matrix. Upon mitochondrial dysfunction, it is accumulated in the cytosol and tends to be imported into the nucleus, activating the transcription of genes encoding mitochondrial chaperones and proteases, proteins of the mitochondrial import machinery and genes involved in metabolism and ROS detoxification.

**Fig. 5:**
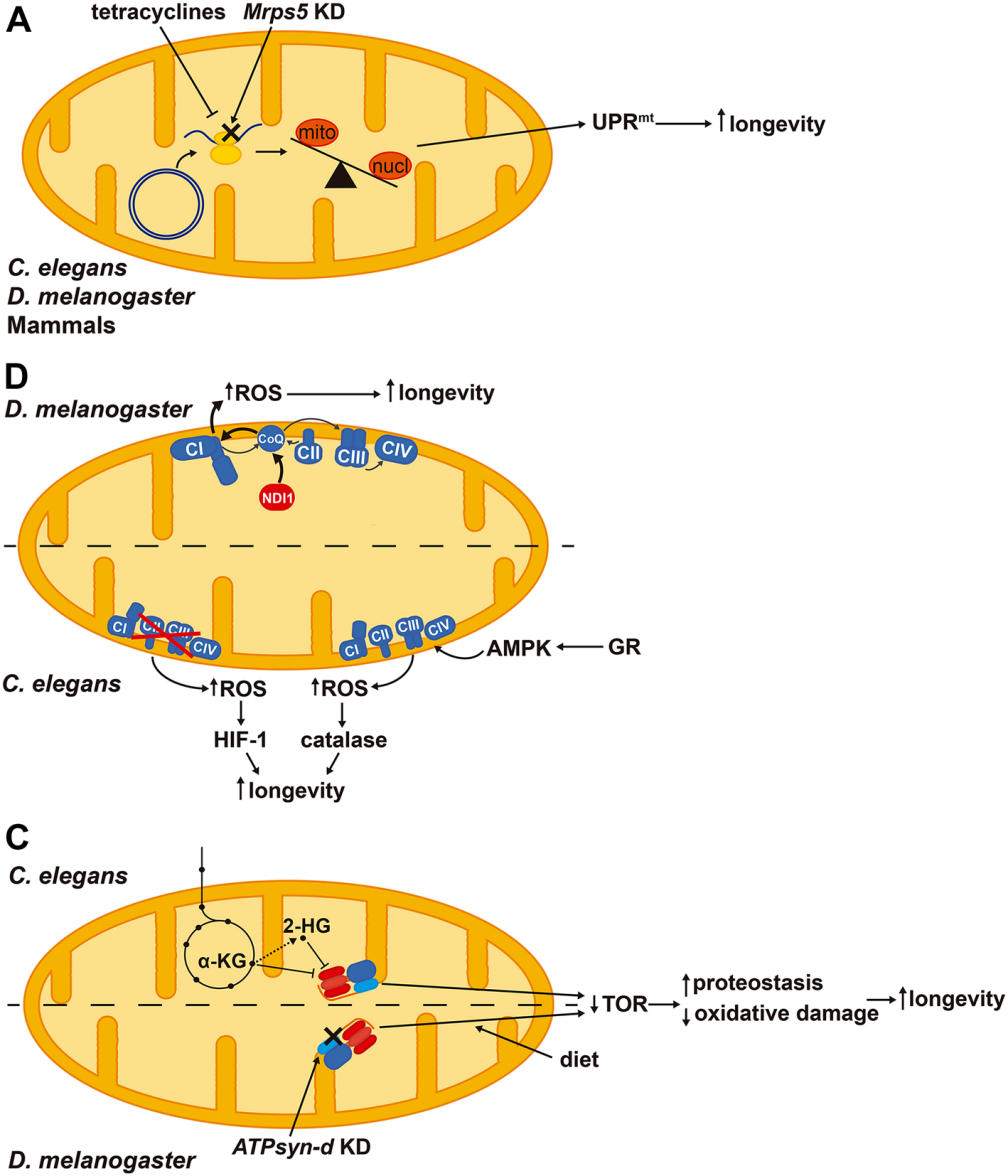
Signaling pathways that arise from mild mitochondrial stresses and contribute to lifespan extension in different model organisms. **a** Activation of the mitochondrial unfolded protein response (UPR^mt^) triggered by an imbalance between mitochondrial- and nuclear-encoded proteins extends longevity. **b** ROS derived from complex I (CI) by over-reduction of CoQ pool and reverse electron transport promote longevity in *Drosophila melanogaster*. Both reduction and enhancement of respiration in *Caenorhabditis elegans* trigger a ROS signal that promotes longevity via HIF-1 (hypoxia-inducible factor 1) or activating antioxidant defenses, respectively. **c** Inhibition of the H^+^-ATP synthase by α-ketoglutarate (α-KG) or 2-hydroxyglutarate (2-HG) increases longevity through repressing TOR (target of rapamycin) signaling and improving proteostasis. Diet composition interacts with the knockdown (KD) of *D. melanogaster* H^+^-ATP synthase subunit d (*ATPsyn-d*) and TOR signaling in extending lifespan. *Mrps5*: mitochondrial ribosomal protein S5, GR: glucose restriction, AMPK: AMP-dependent protein kinase

Activation of UPR^mt^ may extend lifespan. Interestingly, knockdown of *Mrps5* (mitochondrial ribosomal protein S5) and other ribosomal proteins in *C. elegans* impairs the synthesis of mtDNA-encoded ETC subunits and constitutively activates UPR^mt^ extending lifespan [126] (Fig. 5a). In addition, doxycycline—an antibiotic that interferes with mitochondrial translation—also causes an imbalance between mitochondrial- and nuclear-encoded proteins activating UPR^mt^ and extending lifespan in *C. elegans* and muscle fitness in *Drosophila melanogaster* [127] (Fig. 5a). Repletion of NAD^+^ levels in aged mice through the supplementation of its precursor nicotinamide riboside promotes longevity as well as increases the number and regenerative potential of muscle stem cells via UPR^mt^ activation [128]. Importantly, inhibiting this pathway by silencing the prohibitin proteins blunts the pro-longevity effects of nicotinamide riboside [128]. Therefore, activation of UPR^mt^ stress response improves mitochondrial homeostasis by enhancing proteotoxicity resistance and protects stem cells from senescence.

Increasing evidence suggests that the production of low levels of ROS may be protective for the cell, facing against the free-radical theory of aging and the long held supported protective role of antioxidants [21]. While high levels of ROS oxidize and destroy cellular structures [9], low amounts of ROS act as signaling molecules that can activate gene expression programs [4, 129]. Indeed, moderate ROS induce an antioxidant response mediated by Nrf2 [130], as well as mitochondrial biogenesis and expression of OXPHOS genes [131]. ROS have been reported to interact with two pro-longevity pathways in *D. melanogaster*, the UPR^mt^ and antagonizing insulin signaling, which exert cell autonomous and systemic effects, respectively [132]. Interestingly, over-reduction of the coenzyme Q (CoQ) pool in *D. melanogaster* by the expression of NDI1—an alternative oxidoreductase that bypasses ETC complex I (CI)—enhances the production of superoxide radical and extends lifespan [133] (Fig. 5b). This effect is abolished when CI is genetically or pharmacologically inhibited suggesting that reverse electron transport may feed CI with electrons from CoQ to promote ROS production [133]. NDI1 expression, although increases superoxide levels, rescues the mitochondrial defects and the reduced lifespan in SOD2 and PINK1 knockdown flies, pointing out that the site of ROS production is important and might be beneficial for cellular homeostasis [133].

Likewise, reduction of mitochondrial activity has been related to extended lifespan in *C. elegans*. Silencing of subunits of the ETC complexes I, III and IV or *atp-3* (homolog of OSCP) during development promotes longevity [134]. The effect of a mild inhibition of mitochondrial respiration on the extended lifespan appears to occur via increased ROS production, which activates HIF-1 (hypoxia-inducible factor 1)-mediated signaling [135] (Fig. 5b). Interestingly, treatment of worms with low levels of paraquat increases lifespan, while treatment with higher levels of the drug actually reduces lifespan, suggesting the existence of a hormetic response when the generated ROS are mild [135], in agreement with the idea that the amount of generated ROS influence lifespan [136].

The metabolic rate, and hence mitochondrial activity and ROS production have been linked to longevity in two controversial theories of aging [137, 138]. In yeast, an increased respiration rate is related to extended lifespan [139, 140] and calorie restriction—a well-known longevity-promoting intervention—increases mitochondrial respiration [141]. Similarly, calorie restriction extends lifespan in *C. elegans* [142] and in *D. melanogaster* [143] by inducing respiration. In case of the worm, promotion of mitochondrial respiration requires the activation of AMPK (AMP-dependent protein kinase) and also increases ROS production, triggering a cytoprotective antioxidant hormetic response [142] (Fig. 5b). Blunting the yeast TOR signaling pathway either by *tor1* gene deletion or rapamycin treatment enhances the respiratory capacity and chemiosmotic coupling, thereby increasing mitochondrial membrane potential and ROS production [144]. Importantly, uncoupling of respiration in the *tor1*Δ mutants abolishes the pro-longevity effects of TOR inhibition, while triggering mitochondrial ROS production in wild-type strains with menadione is sufficient to extend lifespan [144]. In general, inhibition of mTOR signaling extends lifespan and healthspan in several organisms including mammals, and is believed as a main effector of dietary restriction [145]. Interventions that promote longevity, such as calorie restriction, mild mitochondrial dysfunction and impaired insulin/IGF1-signaling have been related to ROS-mediated signaling [21]. Regarding the link between mitochondrial activity and lifespan, different results have also been reported in mouse. For instance, in the conditional knockout model of the insulin receptor in adipose tissue an upregulated mitochondrial biogenesis and function correlates with an extended longevity apparently unrelated to an enhanced production of ROS [146]. In contrast, the knockout model of the ETC complex IV assembly factor Surf1 (surfeit locus protein 1)—which shows a reduction in cytochrome oxidase activity—is associated with a longer median lifespan and the induction of mitochondrial stress pathways comprising UPR^mt^, Nrf2 and mitochondrial biogenesis [147, 148].

Overall, and in agreement with previous suggestions [136], major thoughts that emerge from the implication of ROS in lifespan extension are that the amount being produced, the species generated, the site of production and the subsequent nuclear response determines ROS influence in lifespan extension.

Besides ROS, mitochondria also produce metabolites that can signal to the rest of the cell [4]. *C. elegans* respiratory mutants with extended lifespan generate high amounts of α-ketoacids [149], molecules structurally related to α-ketoglutarate, a metabolite that promotes longevity [59]. α-ketoglutarate binds to the α and β subunits of the H^+^-ATP synthase and inhibits the complex. By reducing ATP levels, α-ketoglutarate reduces TOR signaling, promoting autophagy and proteostasis that ultimately leads to lifespan extension (Fig. 5c) [59]. 2-hydroxyglutarate, an analog of α-ketoglutarate, also inhibits the H^+^-ATP synthase in *C. elegans*, reducing TOR signaling and extending lifespan [150] (Fig. 5c). Interestingly, treatment of wild-type *C. elegans* with the metabolites generated and extruded by respiratory mutant worms is sufficient to extend lifespan and this occurs at least by HIF-1-mediated signaling [149].

The mitochondrial H^+^-ATP synthase itself has been linked to lifespan because several of its subunits [*atp-3, atp-4, atp-5* and *asb-2*, which, respectively, encode OSCP, F6, d and b subunit homologs and constitute the peripheral stalk of the complex (Fig. 1a)] were identified in RNAi screens for genes promoting longevity in *C. elegans* [134, 151]. Interestingly, a twofold upregulation of the *D. melanogaster* IF1 homolog (CG34423) has been reported in a fly model with extended lifespan [152]. Likewise, silencing of *D. melanogaster ATPsyn-d* (ATP synthase d subunit) reduces TOR signaling and activates genes involved in proteostasis and antioxidant defense, thus promoting a stress resistance phenotype that ultimately leads to lifespan extension [153] (Fig. 5c). Interestingly, this knockdown only promotes longevity when flies are fed with low carbohydrate-to-protein ratio, pointing out that longevity arises from an interaction between diet and the H^+^-ATP synthase [153]. This effect does not seem to be due to electron flux by different supercomplexes [154], because no changes in supercomplex assembly have been reported [153].

**Fig. 6:**
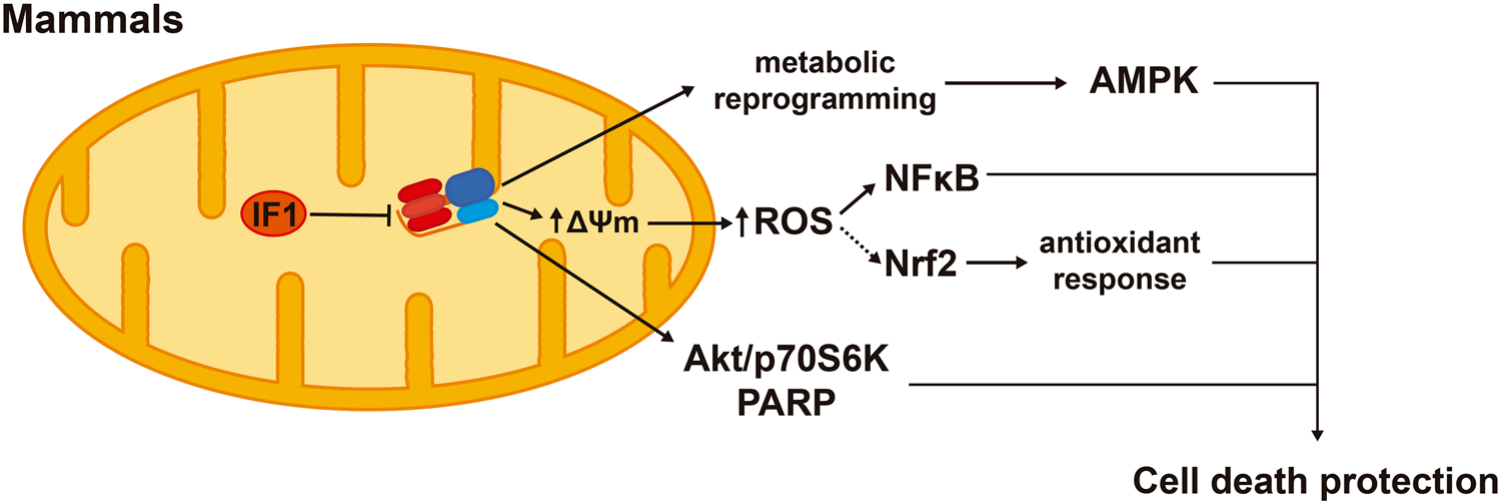
Signaling pathways modulated by IF1 that confer cell death protection. IF1-mediated inhibition of the H^+^-ATP synthase triggers metabolic reprogramming to an enhanced glycolysis and the activation of AMPK (AMP-dependent protein kinase). IF1 inhibition of the synthase also increases ROS production and the ROS-dependent activation of NFκB (nuclear factor kappa B). ROS might also mediate the enhanced Nrf2 [nuclear factor (erythroid-derived 2)-like 2]-guided response that prevents oxidative damage. In neurons, IF1 inhibition of the H^+^-ATP synthase activates Akt (AKT serine/threonine kinase), p70S6K (ribosomal protein S6 kinase, 70 kDa) and PARP (poly(ADP-ribose) polymerase) survival and repair pathways

IF1 overexpression inhibits the H^+^-ATP synthase activity and reprograms energy metabolism to an enhanced glycolysis in cell lines [57] and in in vivo models [42, 58]. The IF1-mediated inhibition of the H^+^-ATP synthase represents a mild mitochondrial dysfunction that confers cytoprotection through the activation of cell survival, antioxidant and repair pathways. In colon cancer cells, IF1 triggers a mild ROS signal that promotes cell survival through NFκB signaling (Fig. 6) [57]. In the in vivo mouse model overexpressing hIF1-H49K in the liver, the transgene triggers the activation of the stress kinase AMPK [42]. In response to an oxidative insult, the transgenic mice deploy an increased antioxidant defense guided by Nrf2 when compared to controls, thus promoting hepatocyte survival [42] (Fig. 6). Likewise, the overexpression in vivo of hIF1-H49K in neurons triggers the activation of AMPK and affords neuroprotection against excitotoxic cell death through NFκB-mediated signaling and Akt (AKT serine/threonine kinase)/p70S6K (ribosomal protein S6 kinase, 70 kDa) and PARP (poly(ADP-ribose) polymerase) repair pathways [58] (Fig. 6). In these mouse models, the overexpression of IF1 is associated with a mild ROS signal as assessed by the carbonylation of cellular proteins and the concurrent activation of AMPK [42, 57, 58] that might reduce mTOR signaling. Hence, we speculate that mitochondrial retrograde signaling by limiting cellular ATP availability and enhancing the production of ROS through the IF1-mediated inhibition of the H^+^-ATP synthase contribute to preserve tissue homeostasis and healthy lifespan.

## Concluding remarks

The H^+^-ATP synthase is a crucial hub that integrates energy metabolism and intracellular signaling pathways that contribute to decide cellular fate. Regulation of the activity of the H^+^-ATP synthase is exerted by its physiological inhibitor IF1. IF1 phosphorylation/ dephosphorylation is regulated during the cell cycle, in hypoxia, by nutrient availability and upon an increase in energy demand of the tissue. Phosphorylation of IF1 by the activity of a mitochondrial cAMP-dependent protein kinase prevents the interaction of the inhibitor with the enzyme allowing ATP synthesis in energized mitochondria or ATP hydrolysis in case that mitochondria become de-energized. When IF1 is bound to the enzyme favors the generation of ROS from the ETC triggering a nuclear response that promotes cell survival by different mechanisms. The outcome of the IF1-mediated signaling depends on the genetic background of the cell. In normal post-mitotic cells, it allows withstanding subsequent detrimental stresses favoring normal tissue function. In malignant cells, IF1 propitiates the acquisition of cancer traits, such as increased proliferation, evasion from cell death and invasion capacity. Actually, IF1 is overexpressed in most prevalent human carcinomas being found essentially in the dephosphorylated state, and hence as an active inhibitor of the H^+^-ATP synthase. In mouse heart under basal conditions, IF1 is present both in the phosphorylated and dephosphorylated states. β-adrenergic stimulation triggers the phosphorylation of IF1 and the activation of ATP production within mitochondria, indicating that there is a fraction of H^+^-ATP synthase in the tissue that is inactive in the handling of ATP but might be active as a “lighthouse” that signals mitohormetic processes by regulating the generation of a mild ROS signal. The identification of the kinase and phosphatase that regulate the activity of IF1, as well as the quantification of dephosphorylated IF1 that is present under basal conditions in different tissues, will contribute to delineate the relevance of the H^+^-ATP synthase in tissue homeostasis. Inhibition of the H^+^-ATP synthase by metabolites and perhaps by other mechanisms contributes to extending lifespan in different organisms through reduced TOR signaling leading to increased proteostasis and reduced oxidative damage. ROS signaling appears to be a common pathway in lifespan extension. Deciphering the precise molecular pathways that are modulated by the H^+^-ATP synthase and contribute to tissue homeostasis and lifespan extension will be of great interest to develop new approaches for therapies in age-related diseases. The generation of mouse models with tissue-specific and regulated expression of IF1 and conditional IF1-knockout models will contribute to unveil the functional role of the H^+^-ATP synthase in cellular physiology and pathophysiology.
